# Wave after wave: determining the temporal lag in Covid-19 infections and deaths using spatial panel data from Germany

**DOI:** 10.1007/s43071-022-00027-6

**Published:** 2022-09-10

**Authors:** Manuela Fritz

**Affiliations:** 1 School of Business, Economics and Information Systems, University of Passau, 94032 Passau, Germany; 2 Department of Economics, Econometrics and Finance, University of Groningen, 9747 AE Groningen, The Netherlands

**Keywords:** Covid-19, Spatio-temporal models, Time lag effects, Spatial spillovers, Spatial Durbin model, Germany, C21, C23, I10, I18

## Abstract

The Covid-19 pandemic requires a continuous evaluation of whether current policies and measures taken are sufficient to protect vulnerable populations. One quantitative indicator of policy effectiveness and pandemic severity is the case fatality ratio, which relies on the lagged number of infections relative to current deaths. The appropriate length of the time lag to be used, however, is heavily debated. In this article, I contribute to this debate by determining the temporal lag between the number of infections and deaths using daily panel data from Germany’s 16 federal states. To account for the dynamic spatial spread of the virus, I rely on different spatial econometric models that allow not only to consider the infections in a given state but also spillover effects through infections in neighboring federal states. My results suggest that a wave of infections within a given state is followed by increasing death rates 12 days later. Yet, if the number of infections in other states rises, the number of death cases within that given state subsequently decreases. The results of this article contribute to the better understanding of the dynamic spatio-temporal spread of the virus in Germany, which is indispensable for the design of effective policy responses.

## Introduction

By the end of 2019, a novel coronavirus, the severe acute respiratory syndrome coronavirus 2, in short Covid-19, was detected in the city of Wuhan in China (Guan et al. 2020; Zhang et al. 2020). What seemed first to cause only a country wide epidemic has fast developed into a worldwide pandemic, which is by now judged as the greatest human challenge since the Second World War. By the time of October 2021, the pandemic has caused about 246 million infections and more than 4.9 million deaths worldwide.

As a policy response, several countries implemented severe lockdowns, including school and store closings, curfews and travel bans to reduce human interaction and thereby the spread of the virus. While aiming to save human life, such restrictions also limit human freedoms. Hence, they require a continuous evaluation of whether they are sufficient to protect vulnerable populations and whether more or less strict measures can and should be considered. One quantitative indicator of policy effectiveness and pandemic severity is the case fatality ratio (WHO 2020; Ioannidis 2021), which is based on the current number of deaths relative to the lagged number of infections. The appropriate length of the time lag to be used, however, is heavily debated (Baud et al. 2020; Kim and Goel 2020). An accurate measure is essential as both, over- and underestimation can have severe consequences, such as not taking the pandemic seriously or causing redundant panic (Kim and Goel 2020).

The literature reflects the uncertainty about the appropriate length. Chrusciel and Szybka (2021), for example, investigate the time lag in several European countries and find that the lag between reported cases and deaths averages around 7 days. Vanella et al. (2020) also investigate the appropriate time lag for European countries to calculate an unbiased case fatality ratio and conclude that a lag between five and ten days should be used. Testa et al. (2020) determine the lag for US counties and find a substantially longer lag. They conclude that deaths often occur two to eight weeks after the onset of the first symptoms. Wilson et al. (2020) determine the case fatality ratio for China and find that a 13-day lag best describes the pattern of the data.

To add to this debate, I provide rigorous evidence for the length of the time lag between a rise in infections and a subsequent rise in death cases using daily panel data for Germany’s 16 federal states from May 2020 to December 2020. In comparison to the studies outlined above, however, I rely on a spatial econometric approach to consider the dynamic spatial spread of the virus. I thereby also add to the literature that analyses the spatial dynamics of Covid-19 and quantifies the spatio-temporal interactions and spillovers of the virus (e.g., Guliyev 2020; Krisztin et al. 2020; Ehlert 2021). Specifically, I estimate different spatial econometric models that allow not only to consider the infections in a given state but also those in neighboring states, so-called spatial lags. Spatial econometric models are useful to model interaction effects between geographical units (Elhorst 2021) and are hence especially useful to model the global-spreading and infectious nature of the coronavirus.

I determine the lag between the wave of infections and the wave of Covid-19 death cases in Germany’s federal states and their geographical spread with four spatial models: I use (1) a model with spatial lags in the independent variables (SLX model), (2) a non-dynamic and (3) a dynamic model that include spatial lags in both, the dependent and independent variable, together with space and time fixed-effects (Spatial Durbin Models with fixed-effects), and (4) a dynamic Spatial Durbin Model with common factors to capture potential strong cross-sectional dependence (i.e., cross-sectional averages instead of time fixed-effects). All of these spatial models allow to derive the direct (same-state) and indirect (other states) effects of infections on death cases. Specifically, the direct effect shows the marginal effect on the number of death cases driven by a change in the number of infections in the own state, while the indirect effect is the marginal effect on the number of death cases driven by an increase in infections in all other states, while both effects taken together form the total effect (Golgher and Voss 2016).

All four spatial econometric models have the advantage that the spillover effects are fully flexible, i.e., they can take any value, which makes them more suitable for economic research focusing on spillover effects in comparison to a Spatial Autoregressive Model (SAR) or Spatial Autoregressive Combined Model (SAC) (Elhorst 2021). Moreover, the two dynamic models allow to differentiate between short-run (SR) and long-run (LR) effects, i.e., is possible to assess whether the effect of an increase in infections on the number of death cases fades out over time, if, for example, effective policies are implemented to protect the most vulnerable.

The remainder of this article proceeds as follows. In Sect. 2, I present the data used for the analysis. In Sect. 3, I outline the empirical strategy and briefly discuss the differences in the spatial econometric models. I present the results in Sect. 4 and conclude in Sect. 5.

## Data

### Daily infections and daily death cases

The main variables used in this article are the number of daily new infections and the number of daily reported death cases due to Covid-19 in Germany at the federal state level. There are in total 16 federal states and the German *Robert Koch Institut* (RKI) provides and updates the respective numbers on a daily basis since the very beginning of the pandemic.^1^ Yet, as the number of cases in the first and second wave are not directly comparable (due to different testing strategies and measures taken), I make use of the data from the period between May 1, 2020 and December 31, 2020, hence dropping observations from the first wave’s peak as well as those that were recorded after the vaccination campaign started. The latter is done to get an estimate of the time lag between infections and deaths without the vaccine being available, hence, an unbiased measure of when the rise of deaths should be expected after a rise of infections.

### Population and geospatial data

To make the numbers of infections and deaths comparable across the different states, I calculate them per 100,000 inhabitants in the respective state. The corresponding population numbers are derived from the *Bundesamt für Kartographie und Geodäsie*^2^ which provides geospatial shape files for Germany, including population numbers for each state. The same source is used to derive the geographical coordinates for each state, which are essential to construct the spatial weight matrix used in spatial models (see Sect. 3).

### Intensive care cases

To be able to derive an unbiased estimate of the time lag between infections and death cases, I control for the number of daily new intensive care (IC) cases per federal state, i.e., the number of patients that have contracted the corona disease and are under intensive care. This variable is also calculated per 100,000 inhabitants. The number of daily new IC cases might be positively related to both variables of interest, the number of infections and deaths. Hence, including the variable in the regression avoids an upward omitted variable bias. Information on the number of patients in IC is provided by the RKI and the German Interdisciplinary Group for Intensive and Emergency Care (DIVI).^3^ The data are available on a daily level and per federal state.

### Temperature data

The medical literature reports that respiratory diseases and infections follow seasonal cycles and are susceptible to temperature (e.g., Shaman et al. 2010; Martinez 2018). Similar patterns have recently been confirmed for the coronavirus. Ma et al. (2020), in their analysis on temperature and humidity effects on Covid-19 deaths in China, find that higher temperatures lead to increases in death cases. Wu et al. (2020) contrarily identify a negative relationship between temperature and new cases and deaths in a study on 166 countries. To control for the potential confounding factor of temperature, I include the daily mean temperature on the state level in my analysis. Data on daily meteorological conditions is received from the German Weather Service (DWD). The DWD provides daily time series data for 538 weather stations across Germany, including the coordinates of each station. I merge each station to the corresponding federal state, using their respective coordinates, and average the mean temperature across all stations.

**Fig. 1:**
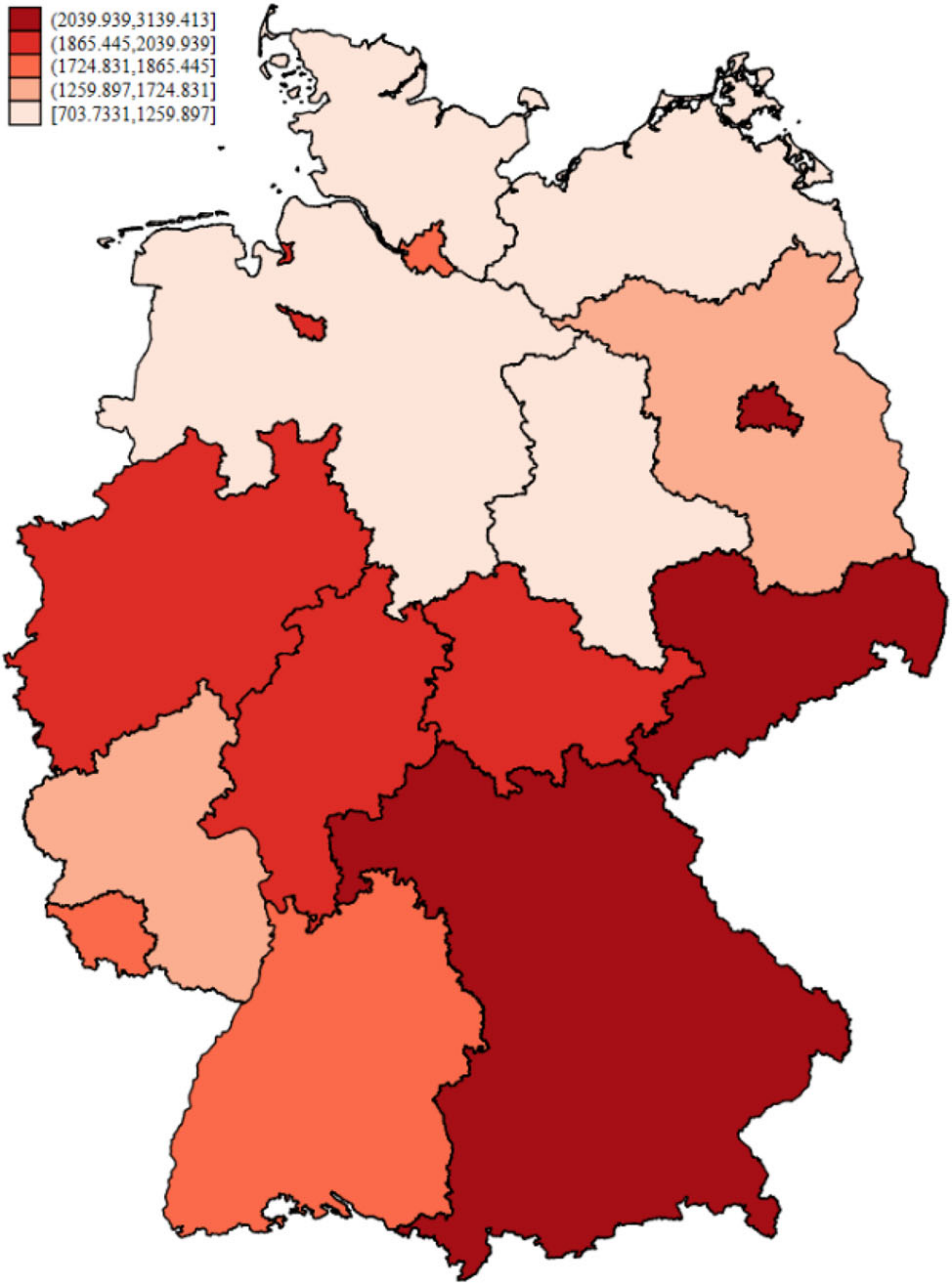
Cumulative Covid-19 infections in Germany. *Notes*: The map shows the cumulative number of Covid-19 infections per 100,000 inhabitants between May 1, 2020 and December 31, 2020 for the 16 federal states in Germany

Figure 1 shows the spatial distribution of the number of infections per 100,000 inhabitants per state cumulated over the observation window from May 1, 2020 to December 31, 2020. The figure shows a clear variation in the number of cumulative Covid-19 cases across all federal states, with states in the south and south-west being the most severely affected. From a first glimpse, cross-sectional dependence seems to be present as there is some clustering of states with similar infection rates.

To confirm the presence of cross-sectional dependence statistically, I use Pesarans’s CD-test for panel data (Pesaran 2004, 2015), which is based on the pairwise correlation coefficients of the different geographical units. I also estimate the standardized cross-sectional exponent $\alpha$ (Bailey et al. 2016) to determine the degree of cross-sectional dependence. The results are shown in Table 1 and confirm the cross-sectional dependence for the number of infections. Cross-sectional dependence is also confirmed for deaths, the number of IC patients and temperature. The correlation is in each case significant at the 1% level and very strong as the exponent $\alpha$ of nearly one indicates. This suggest that a model with common factors instead of time fixed-effects might be better suited to capture the strong cross-sectional dependence (Ciccarelli and Elhorst 2018; Elhorst et al. 2021). Proxying for common factors can be empirically implemented by either cross-sectional averages (pioneered by Pesaran 2006) or principle components (e.g., Bai 2009; Shi and Lee 2017; Bai and Li 2021); the former approach following Pesaran (2006) is applied in this study.

**Table 1:** Pesaran’s CD test for cross-sectional dependence and Bailey et al.’s cross-sectional exponent α

Variable	CD-test	*P* value	Corr	α (s.e.)
Deaths	127.57	0.000	0.744	0.921 (0.06)
Infections	145.18	0.000	0.847	0.917 (1.81)
Intensive care patients	47.94	0.000	0.280	0.919 (0.08)
Mean temperature	165.12	0.000	0.963	0.916 (0.09)

The null-hypothesis of Peseran’s test is weak cross-sectional dependence. *Corr.* is the average pairwise correlation coefficient. Deaths, infections and new intensive care patients are measured per 100,000 inhabitants

## Empirical strategy

To identify the time lag between the number of cases and the number of deaths, I rely on a spatial econometric approach, which allows me to model direct effects within the geographical unit of interest, while accounting for possible interaction effects with neighboring spatial units. Elhorst (2021) differentiates between three types of interactions: (1) exogenous interactions effects (i.e., the independent variable in one spatial unit can affect the dependent variable in other spatial units), (2) endogenous interaction effects (i.e., the dependent variable in one spatial unit can affect the dependent variable in other spatial units), and (3) interaction effects among the error terms (i.e., the error term in one unit can affect the error term in other units). In the modeling approach outlined below, I consider endogenous and exogenous interaction effects.

I use the number of daily deaths per 100,000 inhabitants within each state as dependent variable and regress it on the same-day number of new infections per 100,000 inhabitants in the same state as well as on the number of new infections per 100,000 inhabitants up to 14 days lagged in time in the same state. As explained above, I do control for the number of new patients with the Corona-disease per 100,000 being treated in IC as well as for mean temperature. Both variables also enter the regression with in total 14 time lags. Moreover, I include state fixed-effects to control for all non-time-varying effects that are specific to each state (such as being located at the sea or at the border to a different country) and which might impact the number of daily death cases and infections. Also, day fixed-effects are included to control for time effects affecting all states similarly. This will, for example, control for the effect of the whole country being in lockdown as well as for week-day specific patterns, such as fewer tests during the weekend.

So far, this model corresponds to a ‘standard’ distributed lag model with fixed-effects. To account for the spatial dependence, I further add the spatial components to the model. The full model reads

1 $$\begin{aligned} Deaths_{it} & = \tau Deaths_{it - 1} + \rho \mathop \sum \limits_{j = 1}^{N} w_{ij} Deaths_{jt} + \eta \mathop \sum \limits_{j = 1}^{N} w_{ij} Deaths_{jt - 1} \\ & \quad + \mathop \sum \limits_{k = 0}^{14} \beta_{k} Infections_{it - k} + \mathop \sum \limits_{k = 0}^{14} \theta_{1k} \mathop \sum \limits_{j = 1}^{N} w_{ij} Infections_{jt - k} \\ & \quad + \mathop \sum \limits_{k = 0}^{14} \delta_{k} Temperature_{it - k} + \mathop \sum \limits_{k = 0}^{14} \theta_{2k} \mathop \sum \limits_{j = 1}^{N} w_{ij} Temperature_{jt - k} \\ & \quad + \mathop \sum \limits_{k = 0}^{14} \gamma_{k} Intensive_{it - k} + \mathop \sum \limits_{k = 0}^{14} \theta_{3k} \mathop \sum \limits_{j = 1}^{N} w_{ij} Intensive_{jt - k} \\ & \quad + \alpha_{i} + \sigma_{t} + u_{it} , \\ \end{aligned}$$

where $w_{ij}$ are the elements of the matrix ${\varvec{W}}$, which is a 16 × 16 row-normalized binary contiguity matrix, with its elements being equal to one when state *i* and state *j* are neighboring states and zero otherwise, and all diagonal elements $w_{ij}$ with $i = j$ equal to zero.^4^$\alpha_{i}$ and $\sigma_{t}$ are the federal state and time fixed-effects, respectively, i.e., a binary dummy variable is included for each state *i* and each day *t* (minus one to avoid the dummy variable trap). $Deaths_{it}$, $Infections_{it}$ and $Intensive_{it}$ are the respective numbers of death cases, infections and intensive care patients per 100,000 inhabitants at day *t* in state *i*. $Temperature_{it}$ is the mean temperature at day *t* in state *i.* The subscript *j* always denotes all states excluding state *i*. $u_{it}$ is the error term. $k$ is an index running from 0 to 14 and indicates the respective time lag.

I estimate five different specifications of the outlined model by constraining several of the parameters. In Model (1), I set $\tau = \eta = \rho = \theta_{1k} = \theta_{2k} = \theta_{3k} = 0$, resulting in a standard distributed lag model with spatial and time fixed-effects. With these restrictions, no spatial spillover effects can occur.

In Model (2), I set $\tau = \eta = \rho = 0$, such that the model is the SLX model (spatial lag in independent variables). This model allows only to derive long-run and only local spillover effects, i.e., spatial effects cannot change in size over time and only neighboring states can affect each other—which is, however, an implausible assumption given the global spread of the virus.

In Model (3), therefore, I allow $\rho$ to be different from 0 and thereby for global spillover effects. I only set $\tau = \eta = 0$, leading to the static Spatial Durbin Model with fixed-effects. Finding the parameter $\rho$ to be significantly different from zero implies global spillover effects, since also non-neighboring states can (through other states) affect each other. The static model, however, does not allow to differentiate between short-run and long-run effects, i.e., it does not allow to investigate whether the effects become smaller or larger over time.

Allowing all parameters to differ from zero results in Model (4), the dynamic Spatial Durbin Model with spatial and time fixed effects. As outlined above, adding the dynamic element allows also to differentiate between short-run and long-run effects, i.e., to assess whether the effect size and significance changes over time or remains constant. In Model (5), I drop the time fixed-effects and instead account for common factors by including the cross-sectional averages (following Pesaran 2006) for $Deaths_{it} , Deaths_{it - 1}$, $Infections_{it}$, $Intensive_{it}$ and $Temperature_{it} .$ This approach should be more suitable to account for the strong cross-sectional dependence that was indicated by the high cross-sectional exponent α.

The parameters of the model in Eq. (1) are estimated via Ordinary Least Squares (OLS) if no endogenous interaction effects are included (i.e., in Model (1) and (2)), and by quasi maximum likelihood (QML) if endogenous interaction effects are considered (Model (3)-(5)), since estimating spatial models with endogenous interaction effects via OLS will result in inefficient estimates (LeSage and Pace 2009). The bias correction approach of Yu et al. (2008) is applied in the two dynamic models to yield centered confidence intervals (see also Lee and Yu 2010).

One drawback of the bias-corrected QML estimator is that it will be inconsistent under heteroskedasticity (Bai and Li 2021). While the standard errors for all models are adjusted for possible heteroskedasticity by applying the robust standard error approach proposed by Driscoll and Kraay (1998) (see also Hoechle 2007; Belotti et al. 2017), this will not eliminate the possibility that the QML estimator is not centered around the true value. A quasi-maximum likelihood estimator for dynamic spatial panel data models with common factors that is consistent in the presence of heteroskedasticity has been proposed by Bai and Li (2021), who also show that the bias can reach large magnitudes, especially if the sample size is small. I therefore also present the results of their proposed QML estimator and contrast it with the results from the non-heteroskedasticity robust estimator.^5^

The parameters $\eta ,{ }\rho ,{ }\theta_{1k} ,{ }\theta_{2k}$ and $\theta_{3k}$ jointly determine the spatial interaction effects. The main parameters of interest to determine the time lag are the parameters $\beta_{k}$ and $\theta_{1k}$. However, the point estimates cannot directly be interpreted as the direct effects and the spillover effects (except in the SLX model), but have to be calculated separately. Specifically, as soon as endogenous interaction effects enter the regression, the direct effect is calculated as the mean diagonal element of the models’ N × N matrix of partial derivatives ((1-$\tau$)**I** − (ρ + $\eta$)**W**)^−1^ [β_k_ **I** + **W**θ_k_], where **I** is the identity matrix. The indirect effect is the mean row sum of the non-diagonal elements of the same matrix (LeSage and Pace 2009; Debarsy et al. 2012; Elhorst 2014, 2021). To differentiate between the short- and long-run effects, the respective parameters $\tau$ and $\eta$ have to be set equal to zero.^6^ The corresponding standard errors are computed via Monte Carlo simulations (LeSage and Pace 2009). A complete and detailed overview for the formulas of the marginal effects (for short- and long-run direct and indirect effects) as well as for the respective t-values or standard errors can be found in Elhorst (2014, pp. 25 and 105) and in Belotti et al. (2017, p. 146).

## Results

Table 2 presents the regression coefficients for each of the models outlined above, while Table 3 presents the corresponding (short-run and long-run) marginal direct effects and spillover effects.

**Table 2:** Estimation results of the lag between Covid-19 infections and deaths using different model specifications

	(1)	(2)	(3)	(4)	(5)
	Fixed Effects	SLX	Static SDM with fixed effects	Dynamic SDM with fixed effects	Dynamic SDM with CSA
Deaths t − 1 (τ)				0.178***	− 0.069*
				(0.044)	(0.041)
**W**Deaths t (ρ)			− 0.179***	0.173***	0.076***
			(0.023)	(0.021)	(0.024)
**W**Deaths t − 1 (η)				0.010	0.047
				(0.026)	(0.034)
Infections t	0.017***	0.016***	0.016***	0.016***	0.013***
	(0.003)	(0.002)	(0.002)	(0.002)	(0.002)
Infections t − 1	− 0.002**	− 0.002**	− 0.002**	− 0.005***	− 0.003***
	(0.001)	(0.001)	(0.001)	(0.001)	(0.001)
Infections t − 2	0.000	0.001	0.001*	0.001***	− 0.000
	(0.001)	(0.000)	(0.000)	(0.000)	(0.000)
Infections t − 3	0.000	0.000	0.000	0.000	0.000
	(0.001)	(0.001)	(0.001)	(0.001)	(0.000)
Infections t − 4	0.001	0.000	0.000	0.000	− 0.000
	(0.001)	(0.001)	(0.001)	(0.000)	(0.000)
Infections t − 5	0.001	0.001	0.001	0.001	− 0.001*
	(0.001)	(0.001)	(0.001)	(0.001)	(0.001)
Infections t − 6	− 0.000	0.000	0.000	0.000	− 0.003***
	(0.001)	(0.001)	(0.001)	(0.001)	(0.001)
Infections t − 7	0.000	0.000	0.000	0.000	− 0.001**
	(0.000)	(0.000)	(0.000)	(0.000)	(0.001)
Infections t − 8	0.002***	0.001***	0.001***	0.001**	0.002***
	(0.000)	(0.000)	(0.000)	(0.000)	(0.000)
Infections t − 9	− 0.005***	− 0.005***	− 0.005***	− 0.005***	− 0.002***
	(0.002)	(0.002)	(0.001)	(0.002)	(0.000)
Infections t − 10	− 0.003***	− 0.002***	− 0.002***	− 0.002**	− 0.001*
	(0.001)	(0.001)	(0.001)	(0.001)	(0.000)
Infections t − 11	0.001**	0.002**	0.002***	0.002***	− 0.000
	(0.001)	(0.001)	(0.001)	(0.001)	(0.000)
Infections t − 12	0.006***	0.006***	0.006***	0.006***	0.002***
	(0.001)	(0.001)	(0.001)	(0.001)	(0.000)
Infections t − 13	0.005**	0.005***	0.005***	0.004**	0.003***
	(0.002)	(0.002)	(0.002)	(0.002)	(0.001)
Infections t − 14	0.005***	0.005***	0.005***	0.004***	0.004***
	(0.002)	(0.002)	(0.002)	(0.001)	(0.001)
**W**Infections t		− 0.002***	0.000	0.001**	0.000
		(0.001)	(0.000)	(0.000)	(0.001)
**W**Infections t − 1		0.000	− 0.000	− 0.000	0.000
		(0.000)	(0.000)	(0.001)	(0.000)
**W**Infections t − 2		0.002**	0.002***	0.002***	0.003***
		(0.001)	(0.001)	(0.001)	(0.001)
**W**Infections t − 3		− 0.002***	− 0.002***	− 0.002***	0.001**
		(0.000)	(0.000)	(0.000)	(0.000)
**W**Infections t − 4		− 0.005***	− 0.004***	− 0.004***	− 0.000
		(0.001)	(0.001)	(0.001)	(0.000)
**W**Infections t − 5		− 0.001***	− 0.002***	− 0.001*	0.001
		(0.000)	(0.000)	(0.000)	(0.001)
**W**Infections t − 6		0.000	− 0.000	0.000	0.002***
		(0.000)	(0.001)	(0.000)	(0.001)
**W**Infections t − 7		− 0.000	0.000	− 0.000	0.001
		(0.001)	(0.001)	(0.001)	(0.001)
**W**Infections t − 8		− 0.003***	− 0.002***	− 0.002***	− 0.002***
		(0.001)	(0.001)	(0.001)	(0.000)
**W**Infections t − 9		− 0.001	− 0.001	− 0.001	− 0.000
		(0.001)	(0.001)	(0.001)	(0.000)
**W**Infections t − 10		− 0.001	− 0.000	− 0.000	− 0.000
		(0.001)	(0.001)	(0.001)	(0.000)
**W**Infections t − 11		0.002***	0.003***	0.003***	0.001***
		(0.000)	(0.000)	(0.000)	(0.000)
**W**Infections t − 12		0.001***	0.002***	0.001***	− 0.001**
		(0.000)	(0.000)	(0.000)	(0.000)
**W**Infections t − 13		0.001	0.001**	0.001	− 0.002**
		(0.001)	(0.001)	(0.001)	(0.001)
**W**Infections t − 14		− 0.001**	− 0.000	− 0.000	− 0.002***
		(0.000)	(0.000)	(0.000)	(0.000)
R^2^ (overall, incl. FE)	0.807	0.814	0.814	0.824	0.849
Log Likelihood	2409.24	2489.42	2522.59	2567.47	3285.29
Residual CD-Test	− 9.858	− 9.86	10.71	11.06	− 0.94
Avg. Corr. Coef	− 0.057	− 0.058	0.062	0.065	− 0.005
CS-exponent $\alpha$ of residuals [Conf. Inter]	0.50 [− 0.07; 1.07]	0.34 [− 1.48; 2.16]	0.76 [0.15; 1.07]	0.77 [0.44; 1.11]	0.73 [0.57; 0.90]
LR-test (*p*-value)		(1) versus (2)	(2) versus (3)	(3) versus (4)^1^	(4) versus (5)
		< 0.001	< 0.001	< 0.001	< 0.001
Number of Obs	3920	3920	3920	3904	3904
Number of Fed. States	16	16	16	16	16

Robust Driscoll and Kraay standard errors in parenthesis. ***p < 0.01, **p < 0.05, *p < 0.1. Models (1) and (2) are estimated via OLS. All other models are estimated via Quasi-Maximum Likelihood. The Yu et al. (2008) bias-correction is applied in Model (4) and (5). The Stata command *xsmle* (Belotti et al. 2017) is used to estimate the spatial models. R^2^ statistics show the corrected overall R^2^ including the variation explained by the fixed-effects to be comparable across all five models. ^1^The likelihood ratio test is based on comparing the Log Likelihood between Model (3) and (4) with 2 degrees of freedom. Since this could be considered not fully adequate since the two models are estimated with a different number of observations, I redo the LR test by (i) dropping the (timewise) first observation of Model (3) such that it is also estimated with only 3904 observations and (ii) by including the observation of the last day in April, such that model (4) is estimated with 3920 observations. In both cases, Model (4) is the preferred specification

**Table 3:** Direct effects and spillover effects for infections in the short-run (SR) and long-run (LR)

	SR direct	SR indirect	SR total	LR direct	LR indirect	LR total
*SLX*						
Infections t				0.016***	− 0.002***	
Infections t − 1				− 0.002**	0.000	
Infections t − 2				0.001	0.002**	
Infections t − 3				0.000	− 0.002***	
Infections t − 4				0.000	− 0.005***	
Infections t − 5				0.001	− 0.001***	
Infections t − 6				0.000	0.000	
Infections t − 7				0.000	− 0.000	
Infections t − 8				0.001***	− 0.003***	
Infections t − 9				− 0.005***	− 0.001	
Infections t − 10				− 0.002***	− 0.001	
Infections t − 11				0.002**	0.002***	
Infections t − 12				0.006***	0.001***	
Infections t − 13				0.005***	0.001	
Infections t − 14				0.005***	− 0.001**	
*S-SDM-FE*						
Infections t				0.017***	− 0.002***	0.014***
Infections t − 1				− 0.002**	0.000	− 0.002**
Infections t − 2				0.001	0.002**	0.002***
Infections t − 3				0.000	− 0.002***	− 0.002***
Infections t − 4				0.000	− 0.004***	− 0.004***
Infections t − 5				0.001	− 0.002***	− 0.001
Infections t − 6				0.000	− 0.000	0.000
Infections t − 7				0.000	0.000	0.000
Infections t − 8				0.001***	− 0.002***	− 0.001**
Infections t − 9				− 0.005***	− 0.000	− 0.005***
Infections t − 10				− 0.003***	0.000	− 0.002**
Infections t − 11				0.002***	0.002***	0.004***
Infections t − 12				0.006***	0.001**	0.007***
Infections t − 13				0.005***	0.000	0.005***
Infections t − 14				0.005***	− 0.001**	0.004***
*D-SDM-FE*						
Infections t	0.016***	− 0.002***	0.015***	0.020***	− 0.003***	0.017***
Infections t − 1	− 0.005***	0.001	− 0.004***	− 0.006***	0.001	− 0.005***
Infections t − 2	0.001**	0.002***	0.003***	0.001**	0.002***	0.003***
Infections t − 3	0.000	− 0.002***	− 0.002***	0.000	− 0.003***	− 0.002***
Infections t − 4	0.000	− 0.004***	− 0.003***	0.000	− 0.004***	− 0.004***
Infections t − 5	0.001	− 0.001**	0.000	0.001	− 0.001**	0.000
Infections t − 6	0.000	0.000	0.000	0.000	0.000	0.000
Infections t − 7	0.000	− 0.000	0.000	0.000	− 0.000	0.000
Infections t − 8	0.001***	− 0.002***	− 0.001**	0.001***	− 0.003***	− 0.001**
Infections t − 9	− 0.005***	0.000	− 0.005***	− 0.006***	0.000	− 0.006***
Infections t − 10	− 0.002**	0.000	− 0.002	− 0.002**	0.000	− 0.002
Infections t − 11	0.002***	0.002***	0.004***	0.002***	0.003***	0.005***
Infections t − 12	0.006***	0.000	0.006***	0.007***	0.000	0.007***
Infections t − 13	0.004**	0.000	0.004***	0.005**	0.000	0.005***
Infections t − 14	0.004***	− 0.001***	0.003**	0.005***	− 0.001***	0.004**
*D-SDM-CSA*						
Infections t	0.013***	− 0.001**	0.012***	0.012***	− 0.000	0.012***
Infections t − 1	− 0.003***	0.001	− 0.002***	− 0.003***	0.001	− 0.002***
Infections t − 2	− 0.000	0.003***	0.003***	− 0.000	0.003***	0.003***
Infections t − 3	0.000	0.001*	0.001**	0.000	0.001**	0.001**
Infections t − 4	− 0.000	− 0.000	− 0.001**	− 0.000	− 0.000	− 0.001**
Infections t − 5	− 0.001*	0.001	− 0.000	− 0.001*	0.001	− 0.000
Infections t − 6	− 0.003***	0.002***	− 0.001***	− 0.003***	0.002***	− 0.001***
Infections t − 7	− 0.001**	0.001	− 0.000	− 0.001**	0.001	− 0.000
Infections t − 8	0.002***	− 0.002***	0.000	0.002***	− 0.002***	0.000
Infections t − 9	− 0.002***	− 0.000	− 0.002***	− 0.002***	− 0.000	− 0.002***
Infections t − 10	− 0.001*	− 0.000	− 0.001*	− 0.001*	− 0.000	− 0.001*
Infections t − 11	− 0.000	0.001***	0.000	− 0.000	0.001***	0.000
Infections t − 12	0.002***	− 0.001**	0.001***	0.002***	− 0.001**	0.001***
Infections t − 13	0.003***	− 0.002**	0.000	0.002***	− 0.002**	0.000
Infections t − 14	0.004***	− 0.002***	0.001**	0.003***	− 0.002***	0.001**

Table 3 shows the marginal direct and spillover effects for infections, in the short-run and long-run for every spatial model from day t to day t − 14. Marginal direct (indirect) effects are derived from the mean diagonal (off-diagonal) elements of the respective model’s matrix of partial derivates (LeSage and Pace 2009; Debarsy et al. 2012; Elhorst 2014, 2021). ***p < 0.01, **p < 0.05, *p < 0.1. Standard errors are obtained via Monte Carlo Simulation as described in LeSage and Pace (2009) (see also Belotti et al. 2017; Elhorst 2021). SLX: Spatial lag of X model; S-SDM-FE: Static Spatial Durbin Model with time and spatial fixed effects; D-SDM-FE: Dynamic Spatial Durbin Model with time and spatial fixed effects; D-SDM-CSA: Dynamic Spatial Durbin Model with Cross-Sectional Averages; SR: short-run; LR: long-run

Column (1) in Table 2 displays the results for the standard linear distributed lag model with fixed-effects, in which the point estimates of the parameters can be interpreted directly. It shows, as expected, that the number of daily infections is a strong determinant of the number of deaths cases. An interesting pattern can be observed: the number of the same day infections positively relates to the same day number of deaths cases, while the first lag is negative. This suggests that some form of mortality displacement is present, i.e., a temporary increase in death cases is followed by days with decreased death cases. While this phenomenon has especially been observed during heat waves (see for example Deschênes and Moretti 2009; Karlsson and Ziebarth 2018), studies also investigate this phenomenon in the context of Covid-19 (Michelozzi et al. 2020; Cerqua et al. 2021). Afterwards, the effects become again significantly positive at lag eight, followed once by negative effects at nine and ten days lagged, and then turning once again positive and significant at lag 11–14. Hence, this first basic model suggests that the number of Covid-19 death cases consistently rises after an increase in number of infections after about 11 days. The coefficients for the daily mean temperature are insignificant, while the coefficients for the intensive care variable are first partly negative and become positive after about one week (coefficients for *temperature* and *intensive care cases* are shown in Table 4 in the Appendix). This model, however, does not yet account for spatial interdependence, reflected also in the value of the CD-test for the residuals. It shows that the test statistic does not lie within the interval [− 1.96; 1.96], which is needed to conclude that there is no further cross-sectional dependence in the residuals. At this point it is to mention that the CD-test significantly loses in power as soon as time fixed-effects or common factors are included in a model. Nevertheless, it is used in empirical studies to decide on model fit (see for example Halleck Vega and Elhorst 2016; Ciccarelli and Elhorst 2018), but should always be interpreted carefully and only be considered jointly with other test statistics, namely R-squared, the log-likelihood, and the cross-sectional exponent $\alpha$. The latter supports the rejection of the model as it is not significantly different from 1.

Moving to the SLX model in Column (2) already improves the model fit as the increased values of R-squared and the log-likelihood indicate. It also allows to make a first statement about local spillovers. They show that the number of infections in neighboring states *j* also significantly relates to the death cases in state *i,* yet the coefficients are primarily significantly negative, and only turn positive after a lag of eleven days. Specifically, the spillover effects suggest that an increase in infections in neighboring states leads first to a decrease in own-state death cases with a three to ten-day lag and afterwards to an increase in own-state death cases with an eleven to 13-day lag. In terms of the size, an increase in infections of 1000 per 100,000 inhabitants within a given federal state would lead to about five to six more deaths after 12–13 days, while an increase in infections of 1000 per 100,000 inhabitants in neighboring federal states would lead to one to two more deaths after eleven to 13 days. As before, the direct and indirect effects for temperature are insignificant and those for the intensive care patients support the positive effect after about one week (shown in Table 5 in the Appendix). The residual CD-test statistic jointly with the cross-sectional exponent $\alpha$ being indifferent from 1, however, do again not allow to conclude that this model captures fully the cross-sectional dependence.

Column (3) displays the results for the non-dynamic SDM with fixed effects, which includes now also an endogenous interaction effect. The coefficient *ρ* is significantly negative and several of the $\theta_{1k}$ coefficients also remain negative. Calculating the indirect long-run effects (Table 3) shows that the majority of them are likewise significantly negative, in line with the rather negative than positive spillover effects, as suggested by the results of the SLX model. Exploring the various test statistics reveals that the log-likelihood ratio clearly favors Model (3) over Model (2), yet, the R-squared value is not significantly larger. Moreover, the residual CD statistic is still significant, and $\alpha$ is still not significantly different from 1, which jointly lead to a rejection of the model in terms of fully capturing the spatial dependence.

Introducing the dynamic model in Column (4) shows a somewhat different picture for the *ρ* coefficient, which now turns significantly positive. The likewise positive significant value of $\tau$ suggests serial correlation in the number of deaths. The direct effects are again consistently positive and statistically significant after eleven days, while the indirect effects are again first primarily negative, both in the short- and in the long-run and only turn positive after a lag of eleven days. R-squared becomes somewhat larger in comparison to the static model and also the log-likelihood ratio test rejects the static model in favor of the dynamic model.^7^ Nevertheless, the CD-test statistic still lies outside of the [− 1.96; 1.96] interval and the confidence intervals of the cross-sectional exponent $\alpha$ still include the value 1, making the dynamic SDM with fixed effects also unsuitable to fully capture the strong cross-sectional dependence. Another aspect leading to a rejection of the model is the increasing size (in absolute terms) of both the direct and indirect effect. Given that several federal states implemented cautionary measures to de-link the number of infections and death cases, one would plausibly expect a decrease in the effect sizes over time.

The only model that is well suited to fully capture the cross-sectional dependence in the data is the dynamic SDM with cross-sectional averages presented in Column (5), which aligns with the cross-sectional exponent α of nearly one that was estimated for the raw data. R-squared and the log-likelihood value both take on the largest value in this model and the residual CD-test statistic lies within the [− 1.96; 1.96] interval and is, hence, no longer significant. The average correlation coefficient of the residuals is now close to zero (− 0.005) and the cross-sectional exponent alpha is now significantly different from 1, with the point estimate below the threshold of 3/4, which indicates that common factors have adequately been addressed (Ciccarelli and Elhorst 2018). Moreover, the sum of $\tau , \eta$ and $\rho$ is smaller than 1, which is essential for the model to be stationary. $\tau$ is statistically significant and negative, supporting again the hypothesis of mortality displacement, i.e., days with high numbers of death cases are followed by days with on average lower numbers of death cases.

Several of the $\theta_{1k}$ coefficients as well as the coefficient *ρ* and are statistically significant, *η* remains insignificant, yet, a LR-test rejects that this variable can be dropped from the model. The significant coefficients jointly indicate that global short-run and long-run spillover effects are present. Interestingly, and in contrast to the model in Ciccarelli and Elhorst (2018), the model does only account for the full cross-sectional dependence when cross-sectional averages of both the dependent and the independent variables are included. Including only the cross-sectional averages of the current and lagged independent variable results in a residual CD-test statistic that is still outside the required interval.

As discussed above, the applied QML estimator might be inconsistent in presence of heteroskedasticity. I therefore present the results of the dynamic spatial Durbin model with common factors using the QML estimator proposed by Bai and Li (2021) that explicitly allows for heteroskedasticity in Table 6 in the Appendix. Common factors in this case are modeled as principal components instead of cross-sectional averages. To ease the comparison with the main model in Column (5) of Table 2, which includes five cross-sectional averages, the model estimated with the Bai and Li (2021) estimator includes five common factors (Column (2), Table 6). For further robustness checks, I also present a model with only three common factors (Column (3), Table 6) and a model where the space–time lag is excluded (Column (4), Table 6). The results of these estimations show that some form of bias seems indeed to be present in the main results, primarily for the η, $\beta_{k}$ and $\theta_{1k}$ coefficients. While τ and ρ are fairly similar, the coefficient for the space–time lag (η) turns negative in Columns (2) and (3) of Table 6. Also, the coefficients for infections ($\beta_{k} )$ and spatially weighted infections ($\theta_{1k}$), especially the later lags after 11 or 12 days, are somewhat smaller (in absolute terms) when estimated with the QMLE of Bai and Li (2021). Overall, this implies that the main results presented in Tables 2 and 3 should be cautiously interpreted as upper-bound estimates.

Turning back to the main results, Table 3 shows that the direct effects of increases in the own-state infections are, as expected, positive and significant, which is in line with the hypothesis that increases in infections are followed by increases in deaths. Regarding the size, an increase in the number of infections by 1000 per 100,000 leads to an additional one to three deaths cases in the same state. The time lag between an increase in infections and a subsequent increase in death cases can now be determined at approximately 12 days. Before the 12th lag, the coefficients for both, the direct and the total effect switch between a negative and positive sign, but become consistently positive afterwards in each of the five models.

The estimated indirect effects also remain significantly negative (now even for the longer lags after eleven days) in Model (5), supporting the previous two models in the hypothesis that increases in infection rates in other states decrease own-state death cases. These negative spillover effects might seem counterintuitive at first, but could be an indicator that states choose to implement stricter preventive policy measures in their own state when the infections in other states rise (assuming that own infection rates would remain constant). This finding is also well in line with previous studies. Ehlert (2021), for example, finds that in a given German district the number of deaths and infections shrink with higher numbers in early Covid-19 infections in neighboring districts. Krisztin et al. (2020) also find a temporarily negative degree of global spatial autocorrelation and explain their finding with temporary travel bans to regions with excessive infection rates to prevent transmission to the own country. Nevertheless, these negative spillovers seem to contradict the infectious nature of the virus and should be investigated in further detail in future studies to assess whether the hypothesis of negative spillovers caused by stricter policy measures can be confirmed.

The size of the short-run (SR) and long-run (LR) effects for the preferred Model (5) is now also in line with the theoretical considerations, as they show that the size of all three types of effects becomes smaller in size over time. While this is somewhat blurred in Table 3 given the decimal rounding, taking a closer look reveals that the direct effect after 14 days in the short-run (equal to 0.0036) is reduced by 10% to 0.0033 in the long-run. The same pattern is found for the indirect effect; for example, the indirect short-run effect after 14 days is equal to − 0.0022 but is reduced in absolute terms to − 0.0020. This reduction in absolute size indicates that policy measures that have been taken in the time horizon considered in this study seem to have been effective in protecting the most vulnerable individuals and thereby have slightly weakened the link between the rising number of infections from the rising number of death cases.

The coefficients for temperature remain insignificant in every model and both, the short-run and long-run direct and indirect effects are essentially zero (shown in Table 5 in the Appendix). Hence, in contrast to studies in different settings (Ma et al. 2020; Wu et al. 2020), temperature seems not to affect the number of daily deaths in Germany. Yet, this can at least to some extent be explained by the inclusion of time fixed-effects or common factors, respectively, as they absorb a substantial part of the within-day variation in temperature across states. The number of patients being treated in intensive care seems to relate to the number of deaths (Table 5 in the Appendix), yet no clear positive or negative pattern emerges.

## Conclusion

The severe restrictions and policies that are implemented to limit the dynamic spread of Covid-19 require a continuous evaluation to assess their effectiveness. In this regard, the case fatality ratio is one of the most important quantitative measures (WHO 2020; Ioannidis 2021). Yet, its calculation requires an exact estimate of the time lag between infections and deaths. In this article, I analyze this lag using daily spatial panel data of the 16 German federal states over the period May 2020 to December 2020. My results suggest that the curve of death cases follows the curve of infections with a lag of approximately 12 days.

Moreover, to account for the spatial spread of the virus, I use spatial econometric models that allow for exogenous and endogenous spillover effects between states. I find that only the dynamic Spatial Durbin Model with state-fixed effects and cross-sectional averages can fully capture the strong cross-sectional dependence found in the data. While the overall effect is largely positive across the different models, the direct and indirect effects differ in sign. The direct effects show that an increase in infections within a given state leads to an increase in the number of reported death cases after about 12 days. Contrarily, the indirect effects (spillovers) are significantly negative, indicating that an increase in infections in neighboring states (and via the global spillovers also in all other states) reduces the number of death cases in that given state. This could be explained by preventive measures taken by the state government as soon as they observe rising infections in other states. While this is well line with the findings of earlier studies (Krisztin et al. 2020; Ehlert 2021), future studies will be needed to confirm this hypothesis and to investigate the causal underlying reasons in more detail. Attention needs also to be paid to the fact that the quasi-maximum likelihood estimator applied in this study, which is widely used in the micro- and macro-econometric spatial literature, suffers from the shortcoming of being inconsistent in case of underlying heteroskedasticity (Bai and Li 2021). A comparison of the results with the heteroskedasticity-robust estimator proposed by Bai and Li (2021) revealed that the effect sizes of the increase in death cases after an increase in infections presented and discussed in this article need to be cautiously interpreted as upper-bound estimates. The discussion of the consequences of assuming homoskedasticity in case of underlying heteroskedasticity should therefore receive broader attention in future studies concerning dynamic spatial panel data.

## Appendix

See the Tables 4, 5 and 6.

**Table 4:** Estimation results for intensive care patients and temperature using different model specifications

	(1)	(2)	(3)	(4)	(5)
	Fixed effects	SLX	Static SDM with fixed effects	Dynamic SDM with fixed effects	Dynamic SDM with CSA
Intensive t	0.005	− 0.003	− 0.003	0.009	− 0.014
	(0.015)	(0.012)	(0.012)	(0.013)	(0.017)
Intensive t − 1	− 0.089***	− 0.094***	− 0.096***	− 0.092***	− 0.067***
	(0.021)	(0.025)	(0.023)	(0.025)	(0.022)
Intensive t − 2	− 0.092***	− 0.092***	− 0.093***	− 0.076***	− 0.048***
	(0.034)	(0.031)	(0.029)	(0.028)	(0.016)
Intensive t − 3	− 0.041	− 0.027	− 0.029	− 0.012	− 0.003
	(0.042)	(0.042)	(0.040)	(0.040)	(0.031)
Intensive t − 4	− 0.028**	− 0.028**	− 0.030**	− 0.027*	0.002
	(0.014)	(0.014)	(0.014)	(0.016)	(0.009)
Intensive t − 5	0.002	− 0.015	− 0.016	− 0.014	0.021*
	(0.030)	(0.025)	(0.024)	(0.022)	(0.012)
Intensive t − 6	0.076	0.072*	0.071**	0.071**	0.039***
	(0.047)	(0.038)	(0.034)	(0.030)	(0.014)
Intensive t − 7	0.099**	0.094***	0.085***	0.071**	0.056***
	(0.040)	(0.035)	(0.030)	(0.031)	(0.020)
Intensive t − 8	− 0.008	− 0.000	− 0.000	− 0.013	− 0.015
	(0.021)	(0.019)	(0.019)	(0.022)	(0.022)
Intensive t − 9	0.068**	0.073*	0.071*	0.074**	0.047***
	(0.034)	(0.038)	(0.037)	(0.036)	(0.010)
Intensive t − 10	0.052**	0.052**	0.053**	0.042**	0.059***
	(0.022)	(0.022)	(0.021)	(0.020)	(0.014)
Intensive t − 11	− 0.040**	− 0.049***	− 0.056***	− 0.065***	− 0.005
	(0.015)	(0.018)	(0.019)	(0.020)	(0.016)
Intensive t − 12	− 0.065**	− 0.061**	− 0.059**	− 0.051*	− 0.021
	(0.029)	(0.029)	(0.029)	(0.028)	(0.013)
Intensive t − 13	− 0.017	− 0.005	0.001	0.016	− 0.012
	(0.033)	(0.035)	(0.032)	(0.032)	(0.032)
Intensive t − 14	0.014	0.015	0.015	0.016	− 0.000
	(0.033)	(0.035)	(0.034)	(0.035)	(0.030)
**W**Intensive t		− 0.002***	− 0.013	− 0.015	0.057
		(0.001)	(0.025)	(0.022)	(0.035)
**W**Intensive t − 1		0.000	− 0.005	− 0.005	0.027*
		(0.000)	(0.021)	(0.022)	(0.015)
**W**Intensive t − 2		0.002**	− 0.012	− 0.012	− 0.016
		(0.001)	(0.015)	(0.013)	(0.011)
**W**Intensive t − 3		− 0.002***	0.107***	0.107***	0.072***
		(0.000)	(0.022)	(0.022)	(0.017)
**W**Intensive t − 4		− 0.005***	− 0.005	− 0.022	− 0.023
		(0.001)	(0.015)	(0.015)	(0.018)
**W**Intensive t − 5		− 0.001***	− 0.009	− 0.007	− 0.008
		(0.000)	(0.024)	(0.024)	(0.012)
**W**Intensive t − 6		0.000	− 0.001	0.004	0.005
		(0.000)	(0.031)	(0.029)	(0.012)
**W**Intensive t − 7		− 0.000	− 0.063**	− 0.061**	− 0.051**
		(0.001)	(0.029)	(0.027)	(0.026)
**W**Intensive t − 8		− 0.003***	0.052	0.063*	0.019
		(0.001)	(0.035)	(0.034)	(0.020)
**W**Intensive t − 9		− 0.001	0.025	0.013	− 0.038***
		(0.001)	(0.036)	(0.035)	(0.012)
**W**Intensive t − 10		− 0.001	0.024	0.019	− 0.057*
		(0.001)	(0.017)	(0.013)	(0.035)
**W**Intensive t − 11		0.002***	− 0.041**	− 0.046**	− 0.048***
		(0.000)	(0.020)	(0.018)	(0.012)
**W**Intensive t − 12		0.001***	0.039	0.048	0.010
		(0.000)	(0.026)	(0.029)	(0.010)
**W**Intensive t − 13		0.001	0.149**	0.140**	0.079**
		(0.001)	(0.066)	(0.065)	(0.033)
**W**Intensive t − 14		− 0.001**	0.047	0.022	0.025
		(0.000)	(0.043)	(0.040)	(0.029)
Temperature t	0.004	0.003	0.004	0.004	0.002
	(0.004)	(0.005)	(0.004)	(0.004)	(0.002)
Temperature t − 1	− 0.000	0.000	− 0.000	− 0.001	− 0.000
	(0.002)	(0.002)	(0.002)	(0.002)	(0.001)
Temperature t − 2	− 0.001	− 0.002	− 0.001	− 0.002	0.001
	(0.002)	(0.002)	(0.002)	(0.002)	(0.001)
Temperature t − 3	− 0.002	− 0.001	− 0.001	− 0.000	0.003
	(0.001)	(0.001)	(0.001)	(0.001)	(0.002)
Temperature t − 4	− 0.001	− 0.002	− 0.002	− 0.002	− 0.003**
	(0.001)	(0.002)	(0.001)	(0.002)	(0.001)
Temperature t − 5	− 0.000	− 0.000	− 0.000	0.000	0.001
	(0.001)	(0.001)	(0.001)	(0.001)	(0.001)
Temperature t − 6	0.004	0.005	0.005	0.004	0.001
	(0.004)	(0.004)	(0.004)	(0.004)	(0.001)
Temperature t − 7	0.001	0.001	0.001	− 0.000	− 0.000
	(0.002)	(0.002)	(0.002)	(0.002)	(0.001)
Temperature t − 8	0.002	0.002	0.002	0.002	− 0.000
	(0.001)	(0.001)	(0.001)	(0.001)	(0.001)
Temperature t − 9	− 0.003	− 0.002	− 0.002	− 0.003	0.001
	(0.004)	(0.003)	(0.003)	(0.003)	(0.001)
Temperature t − 10	0.002	0.002	0.002	0.002	0.002
	(0.004)	(0.003)	(0.004)	(0.004)	(0.003)
Temperature t − 11	0.003	0.002	0.002	0.002	0.001
	(0.002)	(0.002)	(0.002)	(0.002)	(0.001)
Temperature t − 12	− 0.002	− 0.003	− 0.003	− 0.004	− 0.002
	(0.002)	(0.003)	(0.003)	(0.003)	(0.002)
Temperature t − 13	0.000	− 0.000	0.000	0.001	0.001
	(0.001)	(0.002)	(0.001)	(0.002)	(0.002)
Temperature t − 14	0.001	0.001	0.001	0.000	0.001
	(0.002)	(0.002)	(0.002)	(0.002)	(0.001)
**W**Temperature t		− 0.000	0.000	− 0.000	0.001
		(0.002)	(0.002)	(0.002)	(0.002)
**W**Temperature t − 1		0.001	0.001	0.001	0.001
		(0.002)	(0.002)	(0.002)	(0.001)
**W**Temperature t − 2		− 0.002	− 0.001	− 0.001	− 0.002
		(0.002)	(0.001)	(0.001)	(0.001)
**W**Temperature t − 3		− 0.002	− 0.002	− 0.002	− 0.003
		(0.003)	(0.002)	(0.003)	(0.002)
**W**Temperature t − 4		0.003	0.003	0.003	0.003*
		(0.003)	(0.002)	(0.003)	(0.002)
**W**Temperature t − 5		− 0.001	0.000	− 0.000	− 0.001
		(0.002)	(0.002)	(0.002)	(0.001)
**W**Temperature t − 6		− 0.003	− 0.003	− 0.003	− 0.001
		(0.003)	(0.003)	(0.003)	(0.001)
**W**Temperature t − 7		− 0.005*	− 0.004**	− 0.004**	− 0.000
		(0.003)	(0.002)	(0.002)	(0.000)
**W**Temperature t − 8		0.004	0.004	0.005	0.001
		(0.004)	(0.004)	(0.004)	(0.001)
**W**Temperature t − 9		− 0.009	− 0.010	− 0.011	− 0.002
		(0.008)	(0.008)	(0.008)	(0.002)
**W**Temperature t − 10		0.005	0.006	0.007	− 0.001
		(0.004)	(0.005)	(0.006)	(0.002)
**W**Temperature t − 11		− 0.001	− 0.000	− 0.001	− 0.001
		(0.002)	(0.002)	(0.002)	(0.001)
**W**Temperature t − 12		0.004	0.003	0.003	0.003
		(0.003)	(0.003)	(0.003)	(0.003)
**W**Temperature t − 13		− 0.003	− 0.003	− 0.004	− 0.002
		(0.004)	(0.004)	(0.005)	(0.002)
**W**Temperature t − 14		0.000	0.000	0.001	− 0.001
		(0.003)	(0.003)	(0.003)	(0.001)
R^2^ (overall, incl. FE)	0.807	0.814	0.814	0.824	0.849
Log likelihood	2409.24	2489.42	2522.59	2567.47	3285.29
Residual CD-Test	− 9.858	− 9.86	10.71	11.06	− 0.94
Avg. Corr. Coef	− 0.057	− 0.058	0.062	0.065	− 0.005
CS-exponent $\alpha$ of residuals [Conf. Inter]	0.50 [− 0.07; 1.07]	0.34 [− 1.48; 2.16]	0.76 [0.15; 1.07]	0.77 [0.44; 1.11]	0.73 [0.57; 0.90]
LR-test (*p*-value)		(1) versus (2)	(2) versus (3)	(3) versus (4)^1^	(4) versus (5)
		< 0.001	< 0.001	< 0.001	< 0.001
Number of obs.	3920	3920	3920	3904	3904
Number of fed. states	16	16	16	16	16

Robust Driscoll and Kraay standard errors in parenthesis. ****p* < 0.01, ***p* < 0.05, **p* < 0.1. Models (1) and (2) are estimated via OLS. All other models are estimated via Quasi-Maximum Likelihood. The Yu et al. (2008) bias-correction is applied in Model (4) and (5). The Stata command *xsmle* (Belotti et al. 2017) is used to estimate the spatial models. R^2^ statistics show the corrected overall R^2^ including the variation explained by the fixed-effects to be comparable across all five models. ^1^The likelihood ratio test is based on comparing the Log Likelihood between Model (3) and (4) with 2 degrees of freedom. Since this could be considered not fully adequate since the two models are estimated with a different number of observations, I redo the LR test by (i) dropping the (timewise) first observation of Model (3) such that it is also estimated with only 3904 observations and (ii) by including the observation of the last day in April, such that model (4) is estimated with 3920 observations. In both cases, Model (4) is the preferred specification

**Table 5:** Direct effects and spillover effects for intensive care patients and temperature, short-run and long-run

	SR direct	SR indirect	SR total	LR direct	LR indirect	LR total
*SLX*						
Intensive t				− 0.003	− 0.005	
Intensive t − 1				− 0.094***	0.010	
Intensive t − 2				− 0.092***	0.007	
Intensive t − 3				− 0.027	0.108***	
Intensive t − 4				− 0.028**	− 0.004	
Intensive t − 5				− 0.015	− 0.008	
Intensive t − 6				0.072*	− 0.011	
Intensive t − 7				0.094***	− 0.079**	
Intensive t − 8				− 0.000	0.051	
Intensive t − 9				0.073*	0.017	
Intensive t − 10				0.052**	0.016	
Intensive t − 11				− 0.049***	− 0.029	
Intensive t − 12				− 0.061**	0.052*	
Intensive t − 13				− 0.005	0.155**	
Intensive t − 14				0.015	0.060	
Temperature t				0.003	− 0.000	
Temperature t − 1				0.000	0.001	
Temperature t − 2				− 0.002	− 0.002	
Temperature t − 3				− 0.001	− 0.002	
Temperature t − 4				− 0.002	0.003	
Temperature t − 5				− 0.000	− 0.001	
Temperature t − 6				0.005	− 0.003	
Temperature t − 7				0.001	− 0.005*	
Temperature t − 8				0.002	0.004	
Temperature t − 9				− 0.002	− 0.009	
Temperature t − 10				0.002	0.005	
Temperature t − 11				0.002	− 0.001	
Temperature t − 12				− 0.003	0.004	
Temperature t − 13				− 0.000	− 0.003	
Temperature t − 14				0.001	0.000	
*S-SDM-FE*						
Intensive t				− 0.002	− 0.012	− 0.014
Intensive t − 1				− 0.096***	0.010	− 0.086***
Intensive t − 2				− 0.094***	0.005	− 0.089***
Intensive t − 3				− 0.033	0.101***	0.067
Intensive t − 4				− 0.029**	0.000	− 0.029
Intensive t − 5				− 0.015	− 0.005	− 0.021
Intensive t − 6				0.073**	− 0.013	0.060*
Intensive t − 7				0.088***	− 0.070**	0.018
Intensive t − 8				− 0.002	0.047	0.045
Intensive t − 9				0.072**	0.012	0.083
Intensive t − 10				0.054**	0.012	0.066**
Intensive t − 11				− 0.055***	− 0.028*	− 0.083***
Intensive t − 12				− 0.060**	0.045*	− 0.015
Intensive t − 13				− 0.007	0.135**	0.128*
Intensive t − 14				0.015	0.042	0.056
Temperature t				0.004	− 0.000	0.003
Temperature t − 1				− 0.000	0.001	0.000
Temperature t − 2				− 0.001	− 0.001	− 0.003
Temperature t − 3				− 0.001	− 0.002	− 0.003
Temperature t − 4				− 0.002	0.003	0.001
Temperature t − 5				− 0.000	0.000	0.000
Temperature t − 6				0.005	− 0.004	0.001
Temperature t − 7				0.001	− 0.004*	− 0.003**
Temperature t − 8				0.002	0.004	0.005
Temperature t − 9				− 0.002	− 0.008	− 0.010
Temperature t − 10				0.002	0.005	0.006
Temperature t − 11				0.003*	− 0.000	0.002
Temperature t − 12				− 0.003	0.003	− 0.000
Temperature t − 13				0.000	− 0.003	− 0.003
Temperature t − 14				0.001	− 0.000	0.001
*D-SDM-FE*						
Intensive t	0.010	− 0.016	− 0.006	0.012	− 0.019	− 0.007
Intensive t − 1	− 0.092***	0.012	− 0.080**	− 0.113***	0.017	− 0.096**
Intensive t − 2	− 0.077***	0.001	− 0.075***	− 0.094***	0.004	− 0.090***
Intensive t − 3	− 0.013	0.097***	0.085**	− 0.016	0.117***	0.101**
Intensive t − 4	− 0.026*	− 0.016	− 0.042*	− 0.032*	− 0.019	− 0.051*
Intensive t − 5	− 0.013	− 0.004	− 0.016	− 0.015	− 0.004	− 0.020
Intensive t − 6	0.073**	− 0.008	0.065**	0.089**	− 0.012	0.078**
Intensive t − 7	0.074**	− 0.065**	0.009	0.091**	− 0.080**	0.011
Intensive t − 8	− 0.014	0.058*	0.044	− 0.017	0.070*	0.052
Intensive t − 9	0.075**	0.002	0.077	0.091**	0.000	0.091
Intensive t − 10	0.043**	0.011	0.054**	0.052**	0.012	0.064**
Intensive t − 11	− 0.064***	− 0.030**	− 0.094***	− 0.078***	− 0.035*	− 0.112***
Intensive t − 12	− 0.050*	0.050*	− 0.000	− 0.061*	0.061*	− 0.000
Intensive t − 13	0.006	0.121**	0.127	0.006	0.145**	0.151
Intensive t − 14	0.014	0.016	0.030	0.017	0.019	0.036
Temperature t	0.004	− 0.001	0.003	0.005	− 0.001	0.004
Temperature t − 1	− 0.001	0.001	0.000	− 0.001	0.001	0.000
Temperature t − 2	− 0.001	− 0.001	− 0.003	− 0.002	− 0.001	− 0.003
Temperature t − 3	− 0.000	− 0.002	− 0.002	− 0.000	− 0.002	− 0.003
Temperature t − 4	− 0.002	0.003	0.001	− 0.002	0.004	0.001
Temperature t − 5	0.000	− 0.000	− 0.000	0.000	− 0.000	− 0.000
Temperature t − 6	0.004	− 0.003	0.001	0.005	− 0.004	0.001
Temperature t − 7	0.000	− 0.003*	− 0.003**	0.000	− 0.004*	− 0.004**
Temperature t − 8	0.001	0.004	0.006	0.002	0.005	0.007
Temperature t − 9	− 0.002	− 0.009	− 0.011	− 0.002	− 0.010	− 0.013
Temperature t − 10	0.002	0.006	0.008	0.002	0.007	0.009
Temperature t − 11	0.002	− 0.001	0.001	0.003	− 0.001	0.001
Temperature t − 12	− 0.004	0.003	− 0.000	− 0.004	0.004	− 0.001
Temperature t − 13	0.001	− 0.003	− 0.003	0.001	− 0.004	− 0.003
Temperature t − 14	0.000	0.000	0.001	0.001	0.000	0.001
*D-SDM-CSA*						
Intensive t	− 0.017	0.055	0.038	− 0.015	0.052	0.037
Intensive t − 1	− 0.068***	0.030**	− 0.038***	− 0.063***	0.026*	− 0.037***
Intensive t − 2	− 0.047***	− 0.013	− 0.060***	− 0.044***	− 0.014	− 0.058***
Intensive t − 3	− 0.003	0.068***	0.065***	− 0.002	0.065***	0.064***
Intensive t − 4	0.001	− 0.022	− 0.021	0.001	− 0.022	− 0.020
Intensive t − 5	0.020*	− 0.009	0.011	0.018*	− 0.008	0.010
Intensive t − 6	0.039***	0.002	0.041**	0.036***	0.004	0.040**
Intensive t − 7	0.056***	− 0.051**	0.005	0.052***	− 0.047*	0.005
Intensive t − 8	− 0.014	0.020	0.006	− 0.013	0.019	0.006
Intensive t − 9	0.047***	− 0.039***	0.008	0.044***	− 0.035***	0.008
Intensive t − 10	0.061***	− 0.058*	0.003	0.057***	− 0.054	0.003
Intensive t − 11	− 0.003	− 0.046***	− 0.049***	− 0.004	− 0.045***	− 0.048***
Intensive t − 12	− 0.021	0.011	− 0.010	− 0.020	0.010	− 0.010
Intensive t − 13	− 0.014	0.075**	0.061	− 0.012	0.072**	0.059
Intensive t − 14	− 0.003	0.025	0.021	− 0.003	0.024	0.021
Temperature t	0.002	0.001	0.003	0.002	0.001	0.003
Temperature t − 1	− 0.000	0.001	0.001	− 0.000	0.001	0.001
Temperature t − 2	0.001	− 0.002	− 0.001	0.001	− 0.002	− 0.001
Temperature t − 3	0.003	− 0.003	0.000	0.003	− 0.003	0.000
Temperature t − 4	− 0.003**	0.003*	− 0.000	− 0.003**	0.003*	− 0.000
Temperature t − 5	0.001	− 0.001	0.000	0.001	− 0.001	0.000
Temperature t − 6	0.001	− 0.001	0.000	0.001	− 0.001	0.000
Temperature t − 7	− 0.000	− 0.000	− 0.000	− 0.000	− 0.000	− 0.000
Temperature t − 8	− 0.000	0.001	0.001	− 0.000	0.001	0.001
Temperature t − 9	0.001	− 0.002	− 0.001	0.001	− 0.002	− 0.001
Temperature t − 10	0.002	− 0.001	0.001	0.002	− 0.001	0.001
Temperature t − 11	0.001	− 0.001	− 0.000	0.001	− 0.001	− 0.000
Temperature t − 12	− 0.002	0.003	0.001	− 0.002	0.003	0.001
Temperature t − 13	0.001	− 0.001	− 0.001	0.001	− 0.001	− 0.001
Temperature t − 14	0.001	− 0.001	0.000	0.001	− 0.001	0.000

Table 5 shows the marginal direct and spillover effects for the number of new intensive care patients and mean temperature, in the short-run and long-run for every spatial model from day t to day t − 14. Marginal direct (indirect) effects are derived from the mean diagonal (off-diagonal) elements of the respective model’s matrix of partial derivates (LeSage and Pace 2009; Debarsy et al. 2012; Elhorst 2014, 2021). ****p* < 0.01, ***p* < 0.05, **p* < 0.1. Standard errors are obtained via Monte Carlo Simulation as described in LeSage and Pace (2009) (see also Belotti et al. 2017; Elhorst 2021). SLX: Spatial lag of X model; S-SDM-FE: Static Spatial Durbin Model with time and spatial fixed effects; D-SDM-FE: Dynamic Spatial Durbin Model with time and spatial fixed effects; D-SDM-CSA: Dynamic Spatial Durbin Model with Cross-Sectional Averages; SR: short-run; LR: long-run

**Table 6:** Estimation results for the dynamic spatial Durbin model using the Bai and Li (2021) heteroskedasticity-robust QMLE

	(1)	(2)	(3)	(3)
	Dynamic SDM with CSA (replication from Column (5), Table 2)	Dynamic SDM with common factors (Bai and Li QMLE) with space–time lag (η)—5 common factors	Dynamic SDM with common factors (Bai and Li QMLE) with space–time lag (η)—3 common factors	Dynamic SDM with common factors (Bai and Li QMLE) without space–time lag (η)—5 common factors
Deaths t − 1 (τ)	− 0.0690	− 0.0697	− 0.0274	− 0.0106
**W**Deaths t (ρ)	0.0758	0.1342	0.1021	0.0940
**W**Deaths t − 1 (η)	0.0466	− 0.0215	− 0.0058	
Infections t	0.0130	− 0.007	− 0.0027	− 0.0038
Infections t − 1	− 0.0028	0.0021	0.0036	0.0035
Infections t − 2	− 0.0004	0.002	0.0029	0.0020
Infections t − 3	0.0000	− 0.0011	− 0.0003	0.0029
Infections t − 4	− 0.0004	− 0.0038	− 0.0086	− 0.0121
Infections t − 5	− 0.0011	0.0029	0.0031	0.0015
Infections t − 6	− 0.0029	0.0024	0.0049	0.0044
Infections t − 7	− 0.0013	0.0000	0.0011	0.0012
Infections t − 8	0.0021	− 0.0075	− 0.0065	− 0.0047
Infections t − 9	− 0.0020	0.0026	0.0030	0.0042
Infections t − 10	− 0.0007	0.0001	0.0010	0.0011
Infections t − 11	− 0.0003	− 0.0003	0.0000	0.0000
Infections t − 12	0.0022	0.0004	0.0002	0.0003
Infections t − 13	0.0026	− 0.0007	− 0.0008	− 0.0008
Infections t − 14	0.0036	0.0002	0.0002	0.0000
**W**Infections t	0.0001	− 0.008	0.0192	− 0.0055
**W**Infections t − 1	0.0004	0.001	0.0106	0.0025
**W**Infections t − 2	0.0032	− 0.0145	0.0408	− 0.0096
**W**Infections t − 3	0.0008	− 0.0104	0.0237	− 0.0073
**W**Infections t − 4	− 0.0005	0.0002	0.0102	0.0068
**W**Infections t − 5	0.0007	0.0144	0.0226	0.0030
**W**Infections t − 6	0.0017	0.0031	0.0187	− 0.0020
**W**Infections t − 7	0.0011	− 0.0033	− 0.0442	− 0.0072
**W**Infections t − 8	− 0.0016	0.0018	0.0956	− 0.0017
**W**Infections t − 9	− 0.0005	− 0.0002	0.1461	− 0.0013
**W**Infections t − 10	− 0.0001	0.0003	0.0176	0.0008
**W**Infections t − 11	0.0007	− 0.0001	0.0048	− 0.0001
**W**Infections t − 12	− 0.0007	− 0.0002	− 0.0008	0.0000
**W**Infections t − 13	− 0.0024	0.0005	0.0087	0.0000
**W**Infections t − 14	− 0.0021	− 0.0002	− 0.0197	− 0.0002
Intensive t	− 0.0143	0.0177	− 0.0027	− 0.0061
Intensive t − 1	− 0.0665	0.008	− 0.0002	− 0.0083
Intensive t − 2	− 0.0484	0.0334	− 0.0042	0.0824
Intensive t − 3	− 0.0033	0.0067	− 0.0020	0.0569
Intensive t − 4	0.0019	− 0.0183	0.0025	0.0095
Intensive t − 5	0.0206	− 0.0024	0.0018	0.0127
Intensive t − 6	0.0388	− 0.0638	0.0015	− 0.0028
Intensive t − 7	0.0561	0.0007	− 0.0028	− 0.0636
Intensive t − 8	− 0.0148	0.0715	0.0006	0.0467
Intensive t − 9	0.0470	0.1776	− 0.0021	0.1556
Intensive t − 10	0.0589	0.0225	− 0.0002	0.0094
Intensive t − 11	− 0.0049	0.0202	0.0022	− 0.0124
Intensive t − 12	− 0.0214	0.008	0.0011	0.0060
Intensive t − 13	− 0.0118	0.0173	− 0.0007	0.0121
Intensive t − 14	− 0.0001	− 0.0189	− 0.0004	− 0.0270
**W**Intensive t	0.0565	− 0.0053	− 0.0062	− 0.0022
**W**Intensive t − 1	0.0266	0.0028	0.0012	0.0038
**W**Intensive t − 2	− 0.0156	0.0591	− 0.0055	0.0147
**W**Intensive t − 3	0.0715	− 0.0222	− 0.0098	0.0024
**W**Intensive t − 4	− 0.0227	− 0.1213	0.0136	− 0.0903
**W**Intensive t − 5	− 0.0081	− 0.1901	0.0014	− 0.1748
**W**Intensive t − 6	0.0045	0.0184	− 0.0036	0.0815
**W**Intensive t − 7	− 0.0509	0.0733	− 0.0061	0.1554
**W**Intensive t − 8	0.0189	0.173	− 0.0019	0.1560
**W**Intensive t − 9	− 0.0377	− 0.0547	− 0.0019	− 0.1869
**W**Intensive t − 10	− 0.0573	0.0009	0.0011	0.0023
**W**Intensive t − 11	− 0.0482	0.0155	0.0000	0.0180
**W**Intensive t − 12	0.0104	− 0.0043	0.0000	− 0.0087
**W**Intensive t − 13	0.0785	− 0.013	0.0001	− 0.0157
**W**Intensive t − 14	0.0253	0.0082	− 0.0004	0.0094
Temperature t	0.0017	− 0.0007	0.0037	− 0.0020
Temperature t − 1	− 0.0002	− 0.0019	0.0088	− 0.0008
Temperature t − 2	0.0010	− 0.0063	0.0600	− 0.0042
Temperature t − 3	0.0033	− 0.0018	0.0032	− 0.0017
Temperature t − 4	− 0.0030	0.0042	− 0.0717	0.0031
Temperature t − 5	0.0014	0.0013	− 0.1655	0.0014
Temperature t − 6	0.0013	0.0028	0.0894	0.0006
Temperature t − 7	− 0.0003	− 0.0037	0.1469	− 0.0035
Temperature t − 8	− 0.0002	− 0.0014	0.0992	− 0.0001
Temperature t − 9	0.0009	− 0.0024	− 0.1327	− 0.0006
Temperature t − 10	0.0021	0.0006	− 0.0228	− 0.0005
Temperature t − 11	0.0008	0.0045	0.0173	0.0022
Temperature t − 12	− 0.0024	0.0027	− 0.0073	0.0015
Temperature t − 13	0.0009	0.0007	− 0.0194	− 0.0009
Temperature t − 14	0.0007	− 0.0006	0.0065	0.0005
**W**Temperature t	0.0010	0.0004	0.0000	− 0.0003
**W**Temperature t − 1	0.0008	− 0.0002	− 0.0016	− 0.0010
**W**Temperature t − 2	− 0.0017	0.0051	0.0032	0.0020
**W**Temperature t − 3	− 0.0031	0.0016	0.0028	0.0029
**W**Temperature t − 4	0.0029	− 0.0011	0.0001	0.0003
**W**Temperature t − 5	− 0.0012	− 0.0019	0.0003	− 0.0003
**W**Temperature t − 6	− 0.0009	− 0.0002	− 0.0021	− 0.0020
**W**Temperature t − 7	− 0.0002	− 0.0011	− 0.0002	0.0006
**W**Temperature t − 8	0.0007	− 0.0001	0.0008	0.0003
**W**Temperature t − 9	− 0.0023	0.0044	0.0031	0.0017
**W**Temperature t − 10	− 0.0011	0.0037	0.0035	0.0026
**W**Temperature t − 11	− 0.0010	− 0.0054	− 0.0047	− 0.0048
**W**Temperature t − 12	0.0032	− 0.0027	− 0.0010	− 0.0011
**W**Temperature t − 13	− 0.0016	− 0.0047	− 0.0012	− 0.0010
**W**Temperature t − 14	− 0.0006	0.003	− 0.0002	0.0005

## Footnotes

1 https://experience.arcgis.com/experience/478220a4c454480e823b17327b2bf1d4/page/Bundesl%C3%A4nder/.

2 See https://sg.geodatenzentrum.de/web_public/gdz/dokumentation/deu/vg250_01-01.pdf for a description of the data set.

3 https://www.intensivregister.de/#/index.

4 ${\varvec{W}}$ can also be defined as inverse distance matrix, where the elements ${w}_{ij}$ are the inverse of the distance between state *i* and state *j.* I also estimated all models using an inverse distance matrix, but this specification performed statistically worse in every model.

5 I would like to thank Kunpeng Li who generously shared his MATLAB code for the heteroskedasticity-robust QMLE with me.

6 In the static Spatial Durbin Model, the parameters τ and η are zero by construction, but the resulting marginal effects are interpreted as long-run marginal effects.

7 The likelihood ratio test is based on comparing the Log Likelihood between Model (3) and (4) with 2 degrees of freedom. Since this could be considered not fully adequate since the two models are estimated with a different number of observations, I redo the LR test by (i) dropping the (timewise) first observation of Model (3) such that it is also estimated with only 3904 observations and (ii) by including the observation of the last day in April, such that model (4) is estimated with 3920 observations. In both cases, Model (4) is the preferred specification.
